# Residency Exposure to Emergency Medical Services Concepts Through Immersion, Interprofessional Collaboration and Assembly Line Education

**DOI:** 10.7759/cureus.20470

**Published:** 2021-12-16

**Authors:** Ayanna Walker, Nubaha Elahi, Maria Tassone, Jonathan Littell, Latha Ganti

**Affiliations:** 1 Emergency Medicine, University of Central Florida College of Medicine, Orlando, USA

**Keywords:** emergency medicine resident, ems education, assembly line education, simulation in medical education, medical education

## Abstract

Introduction: The use of innovative strategies for teaching, such as flipped classroom and assembly line education, has become increasingly popular to engage learners. Residency education has been incorporating these methods to master content, develop critical skills, and improve professionalism.

Methods: We created a three-part immersion experience to teach Emergency Medical Services (EMS) concepts to emergency medicine residents. Residents participated in a mass casualty incident (MCI) in which they were tasked to triage patients and allocate resources in a hospital to treat 11 victims properly. The second portion was to manage a cardiac arrest scenario in the field with the tools our EMS colleagues had available. Lastly, they were asked to create short, high-yield lectures about topics related to EMS.

Results: Pre- and post-test surveys were used to assess the effectiveness of the experience in teaching residents core EMS topics. It was determined that residents not only felt more prepared for an MCI, but they also were more comfortable with their skills as a result of participating in this activity.

Conclusion: Our study further highlights the benefits of non-traditional techniques in residency education. The use of immersion experiences was unique and overall a positive experience for learners. The techniques used in this activity allowed residents to gain confidence in more challenging topics for emergency physicians. This format could be applied to many more topics in the future as an innovative education technique.

## Introduction

There has been an increasing focus on the use of non-traditional teaching techniques to deliver education in a way that is more impactful and beneficial to the learner. A study found that students’ understanding of concepts is more effective when students are actively engaged in learning rather than passive lecture environments [1]. This same study also found that the problem-solving skills of those in a more interactive classroom setting were improved when compared to those in a lecture-based class [1]. Residency education is evolving to incorporate a more active learning environment, and programs seek innovative ways to deliver content. Benefits of engaged learning include student accountability, content mastery, development of critical thinking skills, improvement of interpersonal skills, and improved professionalism [2].

Assembly line education (ALE) has been previously described as an alternative to traditional teaching, creating an active learning environment [3]. It involves rotating learners through a series of activities utilizing flipped classroom pedagogy and hands-on learning. Another high-quality teaching technique involves immersion [4]. Our literature review shows multiple examples of cultural immersion and language immersion and their benefits in medical education [4-7]. We did not, however, find any studies referencing a role-playing immersion experience related to medical education in which the residents function as an ancillary member of the healthcare team. Immersion in this context allows them to better understand the knowledge, constraints, resources, and mindset of the role. It also encourages them to reflect on their own and others’ attitudes towards other professionals’ roles.

Another aspect we wished to incorporate was that of interprofessional collaboration. A study reported on medical student perceptions of a collaborative immersion experience, working alongside different healthcare professionals including nurses, pharmacists, social workers, and respiratory, occupational, and physical therapists [8]. According to the study, this type of learning environment improved understanding of professional roles, helped students understand their own future roles on healthcare teams, and increased awareness of and respect for other professionals, with the potential to change future practice [3].

Utilizing these non-traditional teaching methods, we created a curricular activity to deliver Emergency Medical Services (EMS) concepts to PGY-1, PGY-2, and PGY-3 emergency medicine (EM) residents. Residents participated as EMS personnel involved in mass casualty incident (MCI) triage and management, created and presented high-yield lectures on important EMS topics for EMS personnel and fellow residents, were educated on the functions of the Lifepak® monitor (Physio-Control, Redmond, WA) by EMS personnel, and managed a cardiac arrest simulation as though they were the EMS crew with limited pre-hospital resources.

The purpose of this curricular activity was to educate EM residents on EMS topics through assembly line education and immersion while highlighting the value of interprofessional collaboration and promoting team-based care that benefits the entire healthcare system.

## Materials and methods

Study design

This cohort study was designed to teach EM residents key concepts of pre-hospital care and the management of mass casualty incidents. Participants consisted of a convenience sample of 17 residents who were available to participate in the educational activity during our weekly didactic conference. Participants completed surveys before and after the training program to determine the effectiveness of our novel curriculum in teaching essential principles of pre-hospital care. Our institution issued study exemption (2021-846).

Curricular design

The educational activity consisted of three stations. One station used simulation-based training (SBT) to provide an immersive experience in triaging as pre-hospital personnel during an MCI. The objective of this station was to teach a systematic approach to triage and resource allocation during MCIs. The other station also utilized SBT to simulate a cardiac arrest in a resource-limited environment. The objective of this simulation was to teach residents how to use EMS equipment and practice team-based resuscitation as EMS personnel when responding to critically ill patients. Both SBT stations included a debrief to review the learning objectives and teach key EMS concepts. The last station was a flipped classroom where residents were pre-assigned various EMS topics and gave short, high-yield presentations on their assigned topic. The objective of this station was to become a content expert in core EMS concepts and teach those concepts to the rest of the residency.

This course was held for a total of four hours. Each of the three stations was 60 minutes long. The first 15 minutes of the session was spent pre-briefing all of the participants. The pre-brief focused on the learning objectives for the entire curricular activity, as well as the instructions on how each station would work. The 17 residents were then divided into three groups and rotated between the three stations. The last 45 minutes of the program, the participants returned to an auditorium where they were debriefed as a large group. The debrief focused on triaging and resource allocation during MCIs.

MCI simulation

The MCI SBT took place in the simulation center of a medical school. The scenario included 11 simulated patients who were involved in a motor vehicle collision, which led to a major explosion. The patients consisted of seven mannequins who had realistic moulage and four faculty members who acted as patients. Each of the 11 simulated patients had a description of injuries to help the participants triage and manage the patients in the field. The hallway outside of the simulation center was used to spread the 11 patients on the floor to simulate a post-explosion scene. Two residents roleplayed as EMS personnel and were responsible for triaging all of the patients in five minutes. The other three to four residents role played as emergency physicians who had to prepare the emergency department (ED) for the rapid influx of patients and determine how to allocate all available resources. Three facilitators acted as ED nurses to help residents run the multi-patient simulation. The entire simulation was approximately 30-40 minutes long. Immediately after the simulation, each group was debriefed to discuss how they managed the MCI and to review best practices in MCI triaging and resource allocation.

Pre-hospital team-training simulation

The team-training SBT was held on a staircase within an auditorium to simulate a limited-resource and limited-space environment. The scenario involved an unresponsive patient who was found to be in cardiac arrest on the staircase. A mannequin made to teach BLS was utilized for this simulation. The team used real pre-hospital equipment including a Lifepak to manage the patient. The facilitator for this case used a timer and had each team run the resuscitation for a total of 20 minutes. At the end of the 20 minutes, the team had to decide to terminate care in the field or to transport the patient to the hospital. Once the scenario ended, each group was debriefed on team-based CPR in the field, comparing and contrasting the management of a cardiac arrest in the field versus the emergency department, and the protocols for termination of care in the field.

EMS core concept lecture series

EMS lectures prepared by the residents took place in a large lecture hall in the medical school. This activity used a flipped classroom approach to help residents become content experts in key EMS topics. The format of the lectures was modeled after the American College of Emergency Physicians’ (ACEP) “Drop the Mic” competition. This style encourages speakers to give an engaging and concise lecture on the most high-yield points of a certain topic. Each resident was given five minutes to give a lecture on a pre-assigned EMS topic. The goal was to have each resident master the content and learn in an engaging format from one’s peers.

Program evaluation

Pre- and post-course surveys (Appendix) were created by the EMS director for the EM residency. The goal of the surveys was to measure the effectiveness of the novel course in teaching EMS core concepts to EM residents. A Likert scale was used to measure feedback.

## Results

The primary outcome was overall resident understanding of managing a mass casualty incident and feeling prepared for a mass casualty incident after role immersion by participating in ALE, measured on a 5-point scale. The secondary outcome was knowledge and comfort level in using the Lifepak device by learning from EMS personnel.

Resident characteristics were characterized using descriptive analysis. Responses were analysed using the z-score for two population proportions with a confidence interval (CI) of 95% and a two-tailed hypothesis.

Seventeen residents participated in the immersion experience, of whom 14 completed the pre-intervention survey. Fifty percent (7/14) of respondents were in their first year of training, 36% of residents were in their second year of training (5/14), and 14% of residents were in their third year of training (2/14). Of the 14 respondents, 21% of residents reported prior EMS experience either as an EMT, paramedic, or firefighter. The respondent characteristics can be found in Table 1. Seventeen respondents completed the post-intervention survey. Demographic characteristics were not collected for this group.

**Table 1:** Respondent characteristics EMS, Emergency Medical Services

Characteristic	N (%)
Year of training, n=14	
PGY-1	7 (50)
PGY-2	5 (35.7)
PGY-3	2 (14.3)
Prior EMS experience, n=14	
Yes	3 (21)
No	11 (78.6)

The specific questions for pre- and post-test surveys are summarized in Table 2.

**Table 2:** Questions for pre- and post-test surveys EMS, Emergency Medical Services

Question no.	Question stem
5	I understand the principles of managing a mass casualty incident
6	I feel that I am prepared for a mass casualty incident
7	I prefer a simulated disaster scenario rather than a lecture about mass casualty incidents
9	I know how to use the Lifepak for my everyday ER needs (pacing, defibrillating, obtaining vital signs)
10	I am comfortable using a Lifepak to pace, defibrillate and obtain vitals
11	It is important for me as a physician to have a good relationship with EMS
12	Performing EMS personnel functions in an out-of-hospital cardiac arrest scenario will help me to understand their role better
13	I would like to learn more about EMS
14	How necessary is EMS for pre-hospital care?

The questions that yielded statistically significant results are listed in Table 3.

**Table 3:** Questions that yielded statistically significant results ER, emergency room

Question no.	Question stem
5	I understand the principles of managing a mass casualty incident
6	I feel that I am prepared for a mass casualty incident
9	I know how to use the Lifepak for my everyday ER needs (pacing, defibrillating, obtaining vital signs)
10	I am comfortable using a Lifepak to pace, defibrillate and obtain vitals

Prior to the ALE, 36% of respondents stated they agreed with the statement “I understand the principles of managing a mass casualty incident (MCI),” compared to 94% after the ALE (p=0.0005). Similarly, 21% of respondents reported feeling prepared for a mass casualty incident prior to attending the ALE, compared to 76% after the ALE (p=0.0022). This shows that participating in the assembly line education provided residents with the information and tools they needed to not only understand MCIs, but also improve their comfort level with these situations. There was no statistical significance in regard to preferring a simulated scenario versus lecture. Results of the pre- and post-test surveys are summarized in Table 4, and demonstrated that the exercise did result in a better understanding of MCIs.

**Table 4:** Results of pre- and post-test surveys *Statistically significant

Question no.	Response	Pre-test, (n=14)	Post-test, (n=17)	z score	p value
5	Strongly agree or somewhat agree	5 (36%)	16 (94%)	-3.4618	0.0005*
6	Strongly agree or somewhat agree	3 (21%)	13 (76%)	-3.0518	0.0023*
7	Strongly agree or somewhat agree	11 (79%)	16 (94%)	-1.2849	0.2005
9	Strongly agree	2 (14%)	9 (53%)	-2.2386	0.0251*
10	Strongly agree	2 (14%)	9 (53%)	-2.2386	0.0251*
11	Strongly agree or somewhat agree	14 (100%)	16 (94%)	0.9225	0.3576
12	Strongly agree or somewhat agree	12 (86%)	15 (88%)	-0.2084	0.8337
13	Strongly agree or somewhat agree	13 (93%)	16 (94%)	-0.1422	0.8887
14	Strongly agree or somewhat agree	11 (79%)	16 (94%)	-1.2849	0.2005

## Discussion

There are many studies that demonstrate the effectiveness of providing educational material in innovative manners. We have previously reported on the development of an interactive MCI curriculum [9]. In this study, we implemented assembly line education, interprofessional collaboration, and an immersion experience into the MCI experience.

We utilized the assembly line education format by rotating students through multiple educational exercises to increase active, hands-on learning. Interprofessional collaboration was implemented by having EM residents create educational material for local paramedic students. This allowed them to further understand the EMS scope of practice and gain experience as educators while expanding the paramedic students’ clinical education and experience.

Similar simulation training with an immersion experience has been done to teach other unique skills such as diversity, equity and inclusion (DEI). A recent study used this educational format to incorporate DEI and cultural competency training into their EM residency curriculum [10]. An Australian study used simulation to address issues of health inequalities arising from a lack of cultural sensitivity training [11].

The World Health Organization released the Framework for Action on Interprofessional Education and Collaborative Practice acknowledging that “there is sufficient evidence to indicate that effective interprofessional education enables effective collaborative practice” [12]. Interprofessional educational collaboration is important in all medical fields, but it is especially so for emergency medicine, where nurses, paramedics, EM trainees of all levels and consultants from other fields in medicine work side by side. A survey of medical students found that they had an overall positive attitude towards interprofessional education [13]. However, as with much teaching in emergency medicine, learners do not perceive something as teaching unless it is formally taught or framed as such. A 2018 study confirms that although residents perceived interprofessional communication skills to be “essential for their daily practice,” they did not feel as though their formal education reflected this importance [14]. Interprofessional education not only is crucial for effective communication between teams, but it also directly impacts the quality of care delivered and patient outcomes. A 2013 Cochrane review reported on positive outcomes in emergency department culture and patient satisfaction as well as collaborative team behavior and reduction of clinical error rates for emergency department teams [15].

The current study is unique in that it demonstrates the benefit of immersion experience in medical residency clinical education. Compared to pre-immersion experience, EM residents were more understanding of MCI principles and more confident in their ability to manage an MCI after the immersion activity. Future studies should explore the benefits of immersion experience for further scenarios not frequently encountered in the ED.

## Conclusions

Many studies have shown the benefit of immersive educational experiences. The simulation day we created consisted of three parts: managing a mass casualty incident, flipped classroom teaching of EMS topics, and a pre-hospital team training scenario. The goal was to help residents comprehend the complex management of mass casualty incidents, feel comfortable using Lifepak devices and understand the role of EMS personnel. Based on survey data, it was shown that this was a practical and enjoyable experience for residents. Similar scenarios can be expanded on to further engage residents in learning complex topics in emergency medicine.

## Appendices

Pre-test survey questions

1.     Choose a sequence of 5 numbers or letters that you will remember for the post-survey

2.     Select your PGY level

a.     PGY-1

b.     PGY-2

c.     PGY-3

3.     Have you worked in EMS in some capacity (EMT, paramedic, firefighter)?

a.     Yes

b.     No

4.     I prepared for the EMS ALE with pre-readings

a.     Yes

b.     No

5.     I understand the principles of managing a mass casualty incident

a.     Strongly agree

b.     Somewhat disagree

c.     Neither agree nor disagree

d.     Somewhat agree

e.     Strongly disagree

6.     I feel that I am prepared for a mass casualty incident

a.     Strongly agree

b.     Somewhat disagree

c.     Neither agree nor disagree

d.     Somewhat agree

e.     Strongly disagree

7.     I prefer a simulated disaster scenario rather than a lecture about mass casualty

a.     Strongly agree

b.     Somewhat disagree

c.     Neither agree nor disagree

d.     Somewhat agree

e.     Strongly disagree

8.     I learned through the exercise of creating an EMS mic drop presentation

a.     Strongly agree

b.     Somewhat disagree

c.     Neither agree nor disagree

d.     Somewhat agree

e.     Strongly disagree

9.     I know how to use the Lifepak for my everyday ER needs (pacing, defibrillating)

a.     Strongly agree

b.     Somewhat disagree

c.     Neither agree nor disagree

d.     Somewhat agree

e.     Strongly disagree

10.  I am comfortable using a Lifepak to pace, defibrillate, and obtain vitals

a.     Extremely comfortable

b.     Somewhat comfortable

c.     Neither comfortable nor uncomfortable

d.     Somewhat uncomfortable

e.     Extremely uncomfortable

11.  It is important for me as a physician to have a good relationship with EMS

a.     Strongly agree

b.     Somewhat disagree

c.     Neither agree nor disagree

d.     Somewhat agree

e.     Strongly disagree

12.  Performing EMS personnel functions in an out-of-hospital cardiac arrest scenario will help me to understand their role better

a.     Strongly agree

b.     Somewhat disagree

c.     Neither agree nor disagree

d.     Somewhat agree

e.     Strongly disagree

13.  I would like to learn more about EMS

a.     Strongly agree

b.     Somewhat disagree

c.     Neither agree nor disagree

d.     Somewhat agree

e.     Strongly disagree

14.  How necessary is EMS for pre-hospital care (i.e. very - they should perform interventions prior to arrival, or not necessary - they should just bring the patient to the hospital)?

a.     Extremely necessary

b.     Very necessary

c.     Moderately necessary

d.     Slightly necessary

e.     Not necessary at all

15.  EMS is a valuable medical specialty

a.     Strongly agree

b.     Somewhat disagree

c.     Neither agree nor disagree

d.     Somewhat agree

e.     Strongly disagree

Post-test survey questions

1.     Choose a sequence of 5 numbers or letters that you will remember for the post-survey

2.     I found the EMS ALE applicable to my practice

a.     Strongly agree

b.     Somewhat disagree

c.     Neither agree nor disagree

d.     Somewhat agree

e.     Strongly disagree

3.     I found the EMS ALE to be helpful in providing an EMS personnel perspective for me

a.     Strongly agree

b.     Somewhat disagree

c.     Neither agree nor disagree

d.     Somewhat agree

e.     Strongly disagree

4.     I found it beneficial to have EMS personnel teach about the functions of the Lifepak

a.     Strongly agree

b.     Somewhat disagree

c.     Neither agree nor disagree

d.     Somewhat agree

e.     Strongly disagree

5.     I understand the principles of managing a mass casualty incident

a.     Strongly agree

b.     Somewhat disagree

c.     Neither agree nor disagree

d.     Somewhat agree

e.     Strongly disagree

6.     I feel that I am prepared for a mass casualty incident

a.     Strongly agree

b.     Somewhat disagree

c.     Neither agree nor disagree

d.     Somewhat agree

e.     Strongly disagree

7.     I prefer a simulated disaster scenario rather than a lecture about mass casualty

a.     Strongly agree

b.     Somewhat disagree

c.     Neither agree nor disagree

d.     Somewhat agree

e.     Strongly disagree

8.     I enjoyed having the EMS students in attendance

a.     Strongly agree

b.     Somewhat disagree

c.     Neither agree nor disagree

d.     Somewhat agree

e.     Strongly disagree

9.     I know how to use the Lifepak for my everyday ER needs (pacing, defibrillating)

a.     Strongly agree

b.     Somewhat disagree

c.     Neither agree nor disagree

d.     Somewhat agree

e.     Strongly disagree

10.  I am comfortable using a Lifepak to pace, defibrillate, and obtain vitals

a.     Extremely comfortable

b.     Somewhat comfortable

c.     Neither comfortable nor uncomfortable

d.     Somewhat uncomfortable

e.     Extremely uncomfortable

11.  It is important for me as a physician to have a good relationship with EMS

a.     Strongly agree

b.     Somewhat disagree

c.     Neither agree nor disagree

d.     Somewhat agree

e.     Strongly disagree

12.  Performing EMS personnel functions in an out-of-hospital cardiac arrest scenario will help me to understand their role better

a.     Strongly agree

b.     Somewhat disagree

c.     Neither agree nor disagree

d.     Somewhat agree

e.     Strongly disagree

13.  I would like to learn more about EMS

a.     Strongly agree

b.     Somewhat disagree

c.     Neither agree nor disagree

d.     Somewhat agree

e.     Strongly disagree

14.  How necessary is EMS for pre-hospital care (i.e. very - they should perform interventions prior to arrival, or not necessary - they should just bring the patient to the hospital)?

a.     Extremely necessary

b.     Very necessary

c.     Moderately necessary

d.     Slightly necessary

e.     Not necessary at all

15.  EMS is a valuable medical specialty

a.     Strongly agree

b.     Somewhat disagree

c.     Neither agree nor disagree

d.     Somewhat agree

e.     Strongly disagree

## References

[1] Knight JK, Wood WB (2005). Teaching more by lecturing less. Cell Biol Educ.

[2] Johnson DW, Johnson RT (2002). Learning together and alone: overview and meta‐analysis. Asia Pacific J Educ.

[3] Rosario J, Lebowitz D, Webb AL (2020). Assembly line education: a novel educational technique for today's learners. Cureus.

[4] Crampton P, Dowell A, Parkin C, Thompson C (2003). Combating effects of racism through a cultural immersion medical education program. Acad Med.

[5] Grall K, Panchal A, Chuffe E, Stoneking L (2011). Novel longitudinal curriculum for Spanish immersion education in an emergency medicine residency. ICERI2011 Proc.

[6] Jones ME, Bond ML, Mancini ME (1998). Developing a culturally competent work force: an opportunity for collaboration. J Prof Nurs.

[7] Kavanagh KH (1998). Summers of no return: transforming care through a nursing field school. J Nurs Educ.

[8] House JB, Cedarbaum J, Haque F (2018). Medical student perceptions of an initial collaborative immersion experience. J Interprof Care.

[9] Walker AD, Fusco N, Tsau J, Ganti L (2020). Development of an interactive curriculum and trainee-specific preparedness plan for emergency medicine residents. Int J Emerg Med.

[10] Ward-Gaines J, Buchanan JA, Angerhofer C (2021). Teaching emergency medicine residents health equity through simulation immersion. AEM Educ Train.

[11] Min-Yu Lau P, Woodward-Kron R, Livesay K, Elliott K, Nicholson P (2016). Cultural respect encompassing simulation training: being heard about health through broadband. J Public Health Res.

[12] World Health Organization. (‎2010 (2021). Framework for action on interprofessional education and collaborative practice. https://apps.who.int/iris/handle/10665/70185.

[13] Zechariah S, Ansa BE, Johnson SW, Gates AM, Leo G (2019). Interprofessional education and collaboration in healthcare: an exploratory study of the perspectives of medical students in the United States. Healthcare (Basel).

[14] Olde Bekkink M, Farrell SE, Takayesu JK (2018). Interprofessional communication in the emergency department: residents' perceptions and implications for medical education. Int J Med Educ.

[15] Reeves S, Perrier L, Goldman J, Freeth D, Zwarenstein M (2013). Interprofessional education: effects on professional practice and healthcare outcomes. Cochrane Database System Rev.

